# Endogenous Retroviral Insertions Indicate a Secondary Introduction of Domestic Sheep Lineages to the Caucasus and Central Asia between the Bronze and Iron Age

**DOI:** 10.3390/genes8060165

**Published:** 2017-06-20

**Authors:** Oskar Schroeder, Norbert Benecke, Kai Frölich, Zuogang Peng, Kai Kaniuth, Leonid Sverchkov, Sabine Reinhold, Andrey Belinskiy, Arne Ludwig

**Affiliations:** 1 Leibniz-Institute for Zoo and Wildlife Research, Department of Evolutionary Genetics, Alfred-Kowalke-Straße 17, 10315 Berlin, Germany; oschroeder@izw-berlin.de; 2 German Archaeological Institute, Im Dol 2-6, 14195 Berlin, Germany; norbert.benecke@dainst.de (N.B.); sabine.reinhold@dainst.de (S.R.); 3 Tierpark Arche Warder e.V., Langwedeler Weg 11, 24646 Warder, Germany; kfroelich@arche-warder.de; 4 Southwest University School of Life Sciences, Key Laboratory of Freshwater Fish Reproduction and Development (Ministry of Education), Chongqing 400715, China; pzg@swu.edu.cn; 5 Institut für Vorderasiatische Archäologie, Ludwig-Maximilians-Universität München, Geschwister-Scholl-Platz 1, 80539 Munich, Germany; kaniuth@vaa.fak12.uni-muenchen.de; 6 Institute of Fine Arts, Academy of Sciences of the Republic of Uzbekistan, Afrosiab Street 5/19, Tashkent 100029, Uzbekistan; lsverchkov@mail.ru; 7 Nasledie Ltd., Prospekt Karla Marksa 56, 355017 Stavropol, Russia; nasledie@nasledie.org

**Keywords:** archaeozoology, ancient DNA, endogenous retrovirus, mitochondrial haplotype, retrotype

## Abstract

Sheep were one of the first livestock species domesticated by humans. After initial domestication in the Middle East they were spread across Eurasia. The modern distribution of endogenous Jaagsiekte sheep retrovirus insertions in domestic sheep breeds suggests that over the course of millennia, successive introductions of improved lineages and selection for wool quality occurred in the Mediterranean region and most of Asia. Here we present a novel ancient DNA approach using data of endogenous retroviral insertions in Bronze and Iron Age domestic sheep from the Caucasus and Pamir mountain areas. Our findings support a secondary introduction of wool sheep from the Middle East between the Late Bronze Age and Iron Age into most areas of Eurasia.

## 1. Introduction

Domestic sheep remain the most important livestock in many regions of Asia to this day. First domesticated in the Fertile Crescent around 10,000 BP [1], they were subsequently spread over Eurasia. Of special importance for the northeastern spread of domestic sheep, but also goats and cattle are the Caucasus Mountains [2] and the foothills of the Hindukush and Pamir Mountains in eastern Central Asia [3]. These regions acted as pathways of introduction to northern Central Asia, which was otherwise blocked by inland seas and deserts. Further innovations in sheep breeds and their transmission would also likely have followed these routes.

The Caucasus region is one of the first areas into which domestic sheep were introduced from the Fertile Crescent. In the southern Caucasus, domestic sheep are attested around 8000 BP [4]. In the Ponto-Caspian steppes north of the Caucasus range, sheep are known from Dagestan from the 8th millennium BP [5]. Notably, wild sheep (*Ovis orientalis*) are native to the southern Caucasus, and high genetic diversity in domestic sheep from that region points to likely admixture between wild sheep and early domestic lineages. An early introduction of sheep from the Ponto-Caspian steppes to the western Altai by the Afanasevo culture in the late 6th millennium BP is likely [2].

A second introduction of sheep into Central Asia, specifically to eastern Kazakhstan can be attributed to Bronze Age agro-pastoralist from southern Asia via the western foothills of the Hindukush, Pamir and Tian Shan Mountains by 4500 BP [3,6,7,8,9]. This expansion is probably also responsible for the first introduction of sheep, wheat and bronze to East Asia [7,10]. Later this region was the site of important mountain passes of the Silk Road trading network that allowed trade caravans to enter the Tarim Basin on the eastern side of the mountain ranges. The southern extent of these mountain ranges in Bactria also marked the furthest limits of large Iron Age empires such as the Persian Achaemenid Empire or the empire of Alexander the Great. Consequently, the Pamir Mountains are one of the most important regions for the understanding of cultural exchange and domestic animal movements in Asia.

To gain new insights into domestic sheep population history in Asia, we aimed to establish genetic profiles of sheep remains from three archaeological sites along these focal points of domestic sheep introduction and transmission to northern Central and eastern Asia. The excavations date from the early Bronze Age to the Iron Age, periods when domestic sheep had already been well-established in these regions for millennia.

Previous ancient DNA analyses of archaeological sheep remains have focused on mitochondrial haplotypes [4,11,12,13,14] and compared them to maternal lineages of modern sheep [15,16,17,18,19,20,21]. Of special importance are the mitochondrial haplogroups A and B, which dominate in domestic sheep of eastern Asia and western Eurasia, respectively. This pattern seems to have been present in domestic sheep more than 4000 years ago [4,11,12]. Another genetic marker used to study the population history of domestic sheep, the retrotype, was proposed by Chessa et al. [22]. The retrotype is derived from the presence/absence pattern of four insertionally polymorphic endogenous Jaagsiekte sheep retrovirus insertions (enJSRVs). They showed that primitive North European breeds have high retrotype diversity, based on the frequency of enJSRV-7 and relative rarity of enJSRV-18. In contrast, in sheep breeds from the Mediterranean, the Middle East and Central Asia enJSRV-18 is ubiquitous while enJSRV-7 is rare or absent. The proposed explanation for this distribution is a secondary introduction of improved Middle Eastern wool sheep into these areas [22].

## 2. Materials and Methods

**Figure 1:**
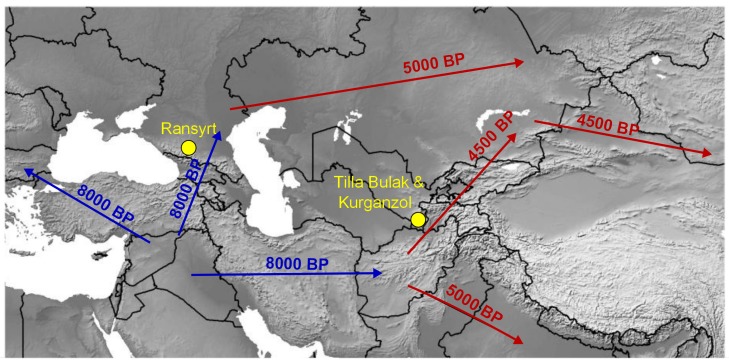
Archaeological sample sites (yellow) and important historical routes for transmission of domestic sheep lineages. Numbers indicate date of first introduction of domestic sheep (blue: sheep first introduced around 8000 BP; red: sheep first introduced around 5000–4000 BP). Map based on Natural Earth.

The sheep bones used in this study come from three archaeological sites (Figure 1). Ransyrt 1 (Stavropol’ region, Russia) is located in the high mountain region of the North Caucasus. The site dates to a short period at the turn of the 17th to the 16th century BC (ca. 3600 BP) [23]. Tilla Bulak (Surkhandarja Province, South Uzbekistan) is a Late Bronze Age settlement site dating to a period of less than 200 years at the beginning of the 2nd millennium BC (ca. 3900 BP) [24]. Kurganzol (Surkhandarja Province, South Uzbekistan) is a Hellenistic fortress which was in use from the late 4th century to the first half of the 2nd century BC (ca. 2300 BP) [25].

Roughly 100 mg of bone powder was taken from the distal right humeri of sheep finds to be certain that all sampled bones belonged to different individuals. The bone powder was further ground to fine size in a Cryo Mill (Retsch, Haan, Germany). Extraction and first PCR set-up were performed in a dedicated ancient DNA lab, while PCR and post-PCR steps were performed in a separate laboratory, according to the recommendations of Cooper and Poinar [26]. DNA was extracted from bone powder according to an established protocol [27]. Of each sample, at least two extractions were made with an additional mock extraction per three extractions. To determine the mitochondrial haplotype, three fragments located in the control region were amplified using a two-step multiplex PCR using the setup of Schröder et al. [14] (Table S1). The product was checked on a 3% agarose gel, sequenced on a 3130XL Genetic Analyzer (Applied Biosystems, Waltham, MA, USA), and compared to known sheep haplotypes [28]. If haplotype determination was successful, the retrotype of a sample was determined by using the same dual PCR setup with novel enJSRV primers (Table S2), but without multiplexing in the first step. Since the original primers used by Arnaud et al. [29] amplified fragments of 500–600 bp, novel primers that amplify 100 bp fragments at the contact between insertion sites and long terminal repeats (LTRs) had to be designed. To check for zygocity, flanking region primers were used to amplify and sequence empty loci. Since the fragmentation of ancient DNA limits the size of amplifiable fragments, it was impossible to check for solo LTRs.

## 3. Results

**Figure 2:**
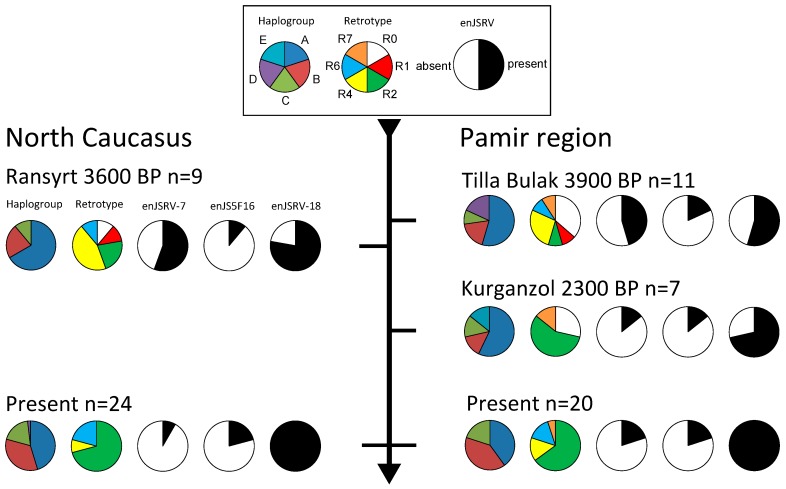
Mitochondrial haplogroup, retrotype and endogenous retrovirus distribution in three archaeological sites from the North Caucasus, the Pamir region and present data from modern breeds of these areas (Present data from Tapio et al. [16] (mitochondrial haplogroups) and Chessa et al. [22] (retroviruses)). Two argali samples from Kurganzol are not included but possessed an argali haplotype and retrotype R0.

Haplotypes and retrotypes were successfully recovered in eleven samples from Tilla Bulak, nine samples from Kurganzol, and nine samples from Ransyrt (Figure 2 and Tables S3–S5).

In all sampled sites mitochondrial haplogroup A was dominant. Haplogroups B and C were also found in each site, while haplogroups D and E were only found in Tilla Bulak and Kurganzol respectively. Additionally, two animals from Kurganzol possessed a haplotype that is otherwise only found in argali (*Ovis ammon*); a wild sheep species that still occurs in Uzbekistan. Since these two individuals also possessed no insertionally polymorphic enJSRVs (R0) and had the largest humerus sizes among all samples, we concluded that they were very likely wild argali and did not include them for further analysis.

As expected, enJSRV-6 was found in each individual from all three sites, including the two wild argali. Animals with retroviral insertions of enJSRV-7, enJS5F16 and enJSRV-18 were found in each site while no animal with enJSRV-8 was detected. For enJSRV-7, heterozygocity could not be determined (see above) and no animal was homozygous for enJS5F16. Only for enJSRV-18, both homozygous and heterozygous individuals were found. A high diversity of retrotypes was present in the Bronze Age sites of Tilla Bulak and Ransyrt, while only three retrotypes were present in Iron Age Kurganzol. While in Bronze Age Tilla Bulak, enJSRV-7 and enJSRV-18 were found in five and six out of eleven individuals respectively, in Iron Age Kurganzol, only a single out of seven domestic individuals possessed enJSRV-7 while only two individuals lacked enJSRV-18. Additionally, all but one of the domestic Kurganzol sheep with enJSRV-18 were homozygous for this insertion. In contrast only two out of six enJSRV-18 carriers were homozygous in Tilla Bulak. In this regard Kurganzol sheep retroviral frequencies resemble a modern Central Asian sample [22] in which enJSRV-18 is ubiquitous while enJSRV-7 is rare. In contrast, the older Tilla Bulak sample shows the larger retrotype diversity reminiscent of modern North European breeds [22]. enJS5F16 was rare but present in both sites, resembling the modern frequency of enJS5F16 in Central Asia [22].

As in Tilla Bulak, enJSRV-7 is much more frequent in the almost contemporaneous Ransyrt site from the northern Caucasus than in a modern sample [22], with five of nine individuals possessing this integration. Similarly, enJSRV-18 is not as common while the frequency of enJS5F16 is comparable.

## 4. Discussion

### 4.1. Mitochondrial Haplogroups

Haplogroup diversity and composition is similar in all sampled sites. However, compared to modern samples from breeds of the respective areas [16] haplogroup A is slightly more common. Additionally, the rare haplogroups D and E were found in the south Central Asian samples. The higher frequencies of haplogroup B in modern breeds [16] of the Caucasus and Central Asia could be a result of a secondary introduction of Middle Eastern breeds, in which haplogroup B is dominating today [16]. Yet in Kurganzol all animals of haplogroup A possessed enJSRV-18 (Table S3).

The presence of argali haplotypes within the Kurganzol sample demonstrates that mitochondrial haplotype data should be used in conjunction with retrotype data, as these wild sheep would have not been recognized with retrotype data alone and would have made the Kurganzol domestic sheep appear more primitive.

### 4.2. Retrotypes

While haplogroup diversity and composition is comparable to modern sheep populations in all sampled sites [16], retrotype diversity is much higher in the Bronze Age sites of Ransyrt and Tilla Bulak than it is in modern samples from the same area [22]. The greater diversity is a result of higher frequencies of enJSRV-7 and lower frequencies of enJSRV-18 compared to modern samples. As noted in this regard they resemble primitive North European breeds [22,30,31]. These breeds are characterized by coarse wool that still possesses larger quantities of guard hair than the wool of economically important merino breeds. Chessa et al. [22] propose that the homogeneity for R2 and R4, that is the near-fixation for enJSRV-18, in most modern Asian and Mediterranean sheep breeds resulted from the secondary introduction of improved wool sheep originating in the Middle East. A hypothesis, which is supported by our data.

An effect of introgression of wild sheep can be dismissed since all wild sheep species lack insertions of the four informative insertionally polymorphous proviruses [22,29,30], and no wild haplotypes were found in clearly domestic sheep. Local selection that favored enJSRV-18 is a possibility, which we cannot exclude. However, local selection would not explain why it would become ubiquitous in almost all modern breeds of Eurasia [22] since the retroviral insertions are, as far as we know, not causally linked with improved wool production.

Originally domestic sheep had been introduced to the Pamir region before 5500 BP, attested in sites such as Sarazm in Tajikistan [3]. As expected, before the proposed secondary introduction, Bronze Age sheep from Tilla Bulak (3900 BP) show high retrotype diversity comparable to relics of older sheep introductions in northern Europe with comparatively high frequencies of enJSRV-7. In contrast, the sample from Iron Age Kurganzol (2300 BP) shows low retrotype diversity, with enJSRV-18 being common and enJSRV-7 being rare. The secondary introduction lineages thus probably reached Bactria and the southern Pamir sometime between 3900 BP and 2300 BP. However, enJSRV-18 was not yet fixed in our admittedly small Iron Age sample (*n* = 7), which might indicate that older lineages were present or contributed to the sheep population of Kurganzol and points to an introduction closer to 2300 BP. A possible context for the introduction of Middle Eastern lineages to southern Central Asia in this timeframe would be the Achaemenid or first Persian Empire that connected these two regions for the first time in a single polity from the 6th to 4th century BC (ca. 2550–2330 BP).

The domestic sheep of the Ransyrt site also seem to be more primitive compared to modern northern Caucasus sheep breeds based on their retrotype diversity and high frequency of enJSRV-7. However, enJSRV-18 is more common compared to the early Bronze Age sample of Tilla Bulak. Due to closer proximity to the origin of the secondary sheep introduction, the improved sheep could have spread to the Caucasus region earlier than to the Pamir region.

As mentioned before in marginal areas of northern Europe older Bronze Age sheep lineages endured until today [22,30,31,32]. Since the improved wool lineages likely originated in the Middle East [30] and were initially more productive in a warm and dry climate, it is possible that they were unable to compete with the cold and wet climate-adapted European Bronze Age lineages in the harsher conditions of northern Europe.

## 5. Conclusions

Our results demonstrate that, in addition to the mitochondrial genome, retroviral insertions can reveal population structure in archaeological material of domestic sheep. The different frequencies of endogenous retroviral insertions enJSRV-7 and enJSRV-18 in Bronze and Iron Age samples point to an influx of new lineages from the Middle East to Central Asia over the course of the third millennium BP. As a direct genetic marker for improved sheep wool is currently not known the retroviral insertion enJSRV-18 associated with modern improved wool breeds is used as a substitute to trace the replacement of older wool sheep lineages. Retroviral data from further sites and time horizons is needed to gain a better understanding of sheep population movements in the Late Bronze and Iron Age.

## Supplementary Materials

The following are available online at www.mdpi.com/2073-4425/8/6/165/s1. Table S1: Mitochondrial CTR-region primers, Table S2: Retroviral primers, Table S3: Mitochondrial haplotype sequences, Table S4: Retroviral sequences, Table S5: Haplo- and retrotypes of sheep samples.
