# Supplementary material for: Endogenous Retroviral Insertions Indicate a Secondary Introduction of Domestic Sheep Lineages to the Caucasus and Central Asia between the Bronze and Iron Age
Source: Genes (Basel). 2017 Jun 20;8(6):165. doi: 10.3390/genes8060165 (PMC5485529; doi:10.3390/genes8060165)
Supplement: Supplementary file 1 [file genes-08-00165-s001.docx]

**Table S1.** Mitochondrial ctr-primers.

| **Primer ID** | **Sequence 5’-3’** | **Reference** |
| --- | --- | --- |
| L15391 | CCACTATCAACACCCAAAG | Cai et al 2011 [1] |
| H15534 | AAGTCCGTGTTGTATGTTTG | Cai et al 2011 [1] |
| OAU15993 | GCATGTAGGGTATTAAACTGCTTGAC | Geörg 2013 [2] |
| OAL16087 | GATCCTTGCRYAGCGGGTTG | Geörg 2013 [2] |
| OAU16068 | CCAYTAGATCACGAGCTTGTTCAC | Geörg 2013 [2] |
| OAL16161 | CTGAAGAAAGAACCAGATGCCTGT | Geörg 2013 [2] |

**Table S2.** Novel retroviral primers.

| **Provirus** | **5’Flank** | **LTRR** | **LTRF** | **3’Flank** |
| --- | --- | --- | --- | --- |
| **enJSRV-6** | CTCTGGGCCCATGACATACT | AGCTCCCAAGACTTAACCCT | CAGGTGCGACTCTTGCTTG | GACACAATTCACTACTTTCAACA |
| **enJSRV-7** | ATCCTCGGCTTGAACGTCA | AGCTCCCAAGACTTAACCCT | CAGGTGCGACTCTTGCTTG | GTGCCACCATTCCAAGAGTC |
| **enJSRV-8** | GGAAACTAGCAAAAAGAGGTTAG | AGCTCCCAAGACTTAACCCT | CTGTGCAGGTGTGACTCTTG | GCCAAACTATTCTTCCCCTTTCT |
| **enJS5F16** | TCGTGTTGAACTCTTTGGCA | AGCTCCCAAGACTTAACCCT | CTGTGCAGGTGTGACTCTTG | GCCCACTCCAGTATTCTTGC |
| **enJSRV-18** | TCGTTTCTTAGGCGCTCTGA | AGCTCCCAAGACTTAACCCT | CAGGTGCGACTCTTGCTTG | TGTGCCCAAGTGCCAGAG |

**Table S3.** Mitochondrial haplotype sequences of two ctr-region fragments in relation to the DomeTree [3] reference sequence

| **Sample** | **15450** | **15459** | **15461** | **15464** | **15466** | **15467** | **15476** | **15484** | **15489** | **15501** | **15508** | **15509** | **15510** | **15512** | **15994** | **16001** | **16003** | **16008** | **16019** | **16020** | **16022** | **16027** | **16028** | **16036** | **16041** | **16042** | **16044** | **16048** | **16052** | **16056** | **16063** | **16064** | **16068** | **16070** | **16096** | **16097** | **16101** | **16128** | **16129** | **16132** | **16133** | **16144** | **16147** | **16156** |
| --- | --- | --- | --- | --- | --- | --- | --- | --- | --- | --- | --- | --- | --- | --- | --- | --- | --- | --- | --- | --- | --- | --- | --- | --- | --- | --- | --- | --- | --- | --- | --- | --- | --- | --- | --- | --- | --- | --- | --- | --- | --- | --- | --- | --- |
| GenBank AF010406.1  Haplotype B1a1b | G | C | A | T | T | T | T | G | T | C | A | A | A | T | C | A | T | G | C | A | T | T | C | G | C | T | T | T | A | C | G | T | C | A | C | A | A | C | T | C | T | C | T | C |
| Ku15 | . | T | G | . | . | . | . | A | . | . | . | . | . | . | . | . | . | . | . | G | C | . | . | A | . | C | . | C | . | . | . | . | . | . | T | G | . | T | . | . | . | . | . | T |
| Ku17 | . | T | . | . | . | . | . | A | . | . | . | . | . | . | . | . | . | . | . | G | C | . | . | A | . | C | . | C | . | . | . | . | . | . | T | G | . | T | . | . | . | . | . | . |
| Ku24 | . | T | . | . | . | . | . | A | . | . | . | . | . | . | . | . | . | . | . | G | C | . | . | A | T | C | . | C | . | . | . | . | . | . | T | G | . | T | . | . | . | . | . | . |
| Ku25 | . | T | . | . | . | . | . | A | . | . | . | . | . | . | . | . | . | . | . | G | C | . | . | A | . | C | . | C | . | . | . | . | . | . | T | G | . | T | . | . | . | . | . | . |
| Ku32 | . | . | . | . | . | . | . | . | . | . | . | . | . | . | T | . | . | . | T | . | . | . | . | . | . | . | C | . | . | . | . | . | . | . | . | . | . | T | . | . | . | . | . | . |
| Ku18 | . | . | . | . | . | . | . | . | . | . | . | G | . | . | . | . | . | A | . | . | . | . | . | A | . | . | C | C | . | . | . | . | . | . | T | G | G | T | . | . | C | . | . | T |
| Ku19 | . | . | . | . | . | . | C | . | . | . | . | G | . | . | . | . | . | A | . | G | . | C | . | A | . | . | . | C | . | . | . | . | . | . | T | G | . | T | . | . | C | . | . | T |
| Ku21 | A | T | . | . | C | C | . | . | C | T | . | . | . | C | . | G | C | . | . | . | . | . | . | A | . | C | . | . | . | . | A | . | . | . | T | G | G | T | C | T | . | T | . | . |
| Ku27 | A | T | . | . | C | C | . | . | C | T | . | . | . | C | N | N | N | N | N | N | N | N | N | N | N | N | N | N | N | N | N | N | N | N | T | G | G | T | C | T | . | T | . | . |
| R261 | . | T | . | . | . | . | . | A | . | . | . | . | . | . | . | . | . | . | . | G | C | . | . | A | . | C | . | C | . | . | . | . | . | . | T | G | . | T | . | . | . | . | . | T |
| R297 | . | T | . | . | . | . | . | A | . | . | . | . | . | . | . | . | . | . | . | G | C | . | . | A | . | C | . | C | . | . | . | . | . | . | T | G | . | T | . | . | . | . | . | . |
| R406 | . | T | . | . | . | . | . | A | . | . | . | . | . | . | . | . | . | . | . | G | C | . | . | A | . | C | . | C | . | . | . | . | . | . | T | G | . | T | . | . | . | . | . | . |
| R431 | . | T | G | . | . | . | . | A | . | . | . | . | . | . | . | . | . | . | . | G | C | . | . | A | . | C | . | C | . | . | . | . | . | . | T | G | . | T | . | . | . | . | . | . |
| R599 | . | T | . | . | . | . | . | A | . | . | . | . | . | . | . | . | . | . | . | G | C | . | . | A | . | C | . | C | . | . | . | . | A | C | T | G | . | T | . | . | . | . | C | . |
| R576 | . | T | . | . | . | . | . | A | . | . | . | . | . | . | . | . | . | . | . | G | C | . | . | A | . | C | . | C | . | . | . | . | . | . | T | G | . | T | . | . | . | . | . | . |
| R411A | . | . | . | . | . | . | . | . | . | . | . | . | . | . | . | . | . | . | . | . | . | . | . | . | . | . | . | . | . | . | . | . | . | . | . | . | . | T | . | . | . | . | . | . |
| R411B | . | . | . | . | . | . | . | . | . | . | . | . | . | . | . | . | . | . | . | . | . | . | . | . | . | . | C | . | . | . | . | . | . | . | . | . | . | T | . | . | . | . | . | . |
| R276 | . | . | . | . | . | . | . | . | . | . | . | G | . | . | . | . | . | A | . | . | . | . | . | A | . | . | C | C | . | . | . | . | . | . | T | G | G | T | . | . | C | . | . | . |
| TB10-709 | . | T | . | . | . | . | . | A | . | . | . | . | . | . | . | . | . | . | . | G | C | . | . | A | . | C | . | C | C | . | . | . | . | . | T | G | . | T | . | . | . | . | . | . |
| TB10-683 | . | T | . | . | . | . | . | A | . | . | . | . | . | . | . | . | . | . | . | G | C | . | . | A | . | C | . | C | . | . | . | . | . | . | T | G | . | T | . | . | . | . | . | . |
| TB08-310 | . | T | . | . | . | . | . | A | . | . | G | . | . | . | . | . | . | . | . | G | C | . | T | A | . | C | . | C | . | . | . | . | T | . | T | G | . | T | . | . | . | . | . | . |
| TB10-689 | . | T | . | C | . | . | . | A | . | . | . | . | . | . | . | . | . | . | . | G | C | . | . | A | . | C | . | C | . | . | . | . | T | . | T | G | . | T | . | . | . | . | . | . |
| TB10-831 | . | T | . | . | . | . | . | A | . | . | . | . | . | . | . | . | . | . | . | G | C | . | . | A | . | C | . | C | . | . | . | . | . | . | T | G | . | T | . | . | . | . | . | . |
| TB08-266 | . | T | . | . | . | . | . | A | . | . | . | . | . | . | . | . | . | . | . | G | C | . | . | A | . | C | . | C | . | . | . | . | . | . | T | G | . | T | . | . | . | . | . | . |
| TB10-897 | . | . | . | . | . | . | . | . | . | . | . | . | . | . | . | . | . | . | . | . | . | . | . | . | . | . | . | . | . | . | . | . | . | . | - | - | . | T | . | . | . | . | . | . |
| TB10-792 | . | . | . | . | . | . | . | . | . | . | . | . | . | . | . | . | . | . | . | . | . | . | . | . | . | . | . | . | . | T | . | . | . | . | . | . | . | T | . | . | . | T | . | . |
| TB09-714 | . | . | . | . | C | . | . | . | . | . | . | G | . | . | . | . | . | A | . | . | . | . | . | A | . | . | C | C | . | . | . | . | . | . | T | G | G | T | . | . | C | . | . | T |
| TB10-657 | . | . | . | . | . | . | . | . | . | . | . | . | -- | C | . | . | . | A | . | G | . | . | . | A | . | C | . | C | . | . | . | C | . | . | . | . | . | T | C | . | . | . | . | . |
| TB09-716 | . | . | . | . | . | . | . | . | . | . | . | . | . | C | . | . | . | A | . | G | . | . | . | A | . | C | . | C | . | . | . | C | . | . | T | G | . | T | C | . | . | . | . | . |

**Table S4.** Sequences of the flanking regions, 6 bp- duplication (underscore), and LTRs (bold) of the five amplified proviruses [4,5] using the primers of Table S2.

| **Provirus** | **Position** | **Sequence** |
| --- | --- | --- |
| enJSRV-6 | 5’Flank-LTR | AGTTATTCTTTTGATTGT**TGCGGGGGACGACCCGTGA** |
|  | LTR-3’Flank | **TGCTGGCCGCGGCA**GATTGTCATTCCTGAATATAATCTGA |
|  | empty locus | - |
| enJSRV-7 | 5’Flank-LTR | AGGCAGTGCAGGGAAAACAGGTTCCTCTG**TGCGGGGGACGACCCGTAA** |
|  | LTR-3’Flank | **TGCTGGCCGCGGCA**CCTCTGGAATGGACTGACACATCTGGGG |
|  | empty locus | AGGCAGTGCAGGGAAAACAGGTTCCTCTGGAATGGACTGACACATCTGGGG |
|  |  |  |
| enJSRV-8 | 5’Flank-LTR | GTATTTAAGGTAAGG**TGCGGGGGACTGCCCGTGA** |
|  | LTR-3’Flank | **CTTGTGCTGGCCGCGGCA**GTAAGGTTGTTCAGAT |
|  | empty locus | GTATTTAAGGTAAGGTTGTTCAGAT |
| enJS5F16 | 5’Flank-LTR | GTCTGCCAGGCTCCTCTGTCC**TGCGGGGGACGACCCGTGA** |
|  | LTR-3’Flank | **CTTGTGCTGGCCGCGGCA**CTGTCCATGGGATTTCCCAG |
|  | empty locus | GTCTGCCAGGCTCCTCTGTCCATGGGATTTCCCAG |
| enJSRV-18 | 5’Flank-LTR | GCCTGATTGCATTTAGGAAG**TGCGGGGGACGACCCGTGA** |
|  | LTR-3’Flank | **TGCTGGCCGCGGC**AGGAAGAGCTGTGGGCTGGCTCTGGG |
|  | empty locus | GCCTGATTGCATTTAGGAAGAGCTGTGGGCTGGCTCTGGG |

**Table S5.** Haplo- and retrotypes of sheep samples.

| **Sample** | **Locality** | **Age** | **Haplogroup** | **V6** | **V7** | **V8** | **F16** | **V18** | **Retrotype** |
| --- | --- | --- | --- | --- | --- | --- | --- | --- | --- |
| Ku15 | Kurgansol | ca 2300 BP | A | +/+ | - | - | - | +/+ | R2 |
| Ku17 | Kurgansol | ca 2300 BP | A | +/+ | - | - | - | +/+ | R2 |
| Ku24 | Kurgansol | ca 2300 BP | A | +/+ | - | - | - | +/+ | R2 |
| Ku25 | Kurgansol | ca 2300 BP | A | +/+ | - | - | - | +/+ | R2 |
| Ku32 | Kurgansol | ca 2300 BP | B | +/+ | + | - | +/- | +/- | R7 |
| Ku18 | Kurgansol | ca 2300 BP | C | +/+ | - | - | - | - | R0 |
| Ku19 | Kurgansol | ca 2300 BP | E | +/+ | - | - | - | - | R0 |
| Ku21 | Kurgansol | ca 2300 BP | wild argali | +/+ | - | - | - | - | R0 |
| Ku27 | Kurgansol | ca 2300 BP | wild argali | +/+ | - | - | - | - | R0 |
| R261 | Ransyrt | ca 3600 BP | A | +/+ | - | - | - | +/- | R2 |
| R297 | Ransyrt | ca 3600 BP | A | +/+ | + | - | - | - | R1 |
| R406 | Ransyrt | ca 3600 BP | A | +/+ | + | - | - | +/- | R4 |
| R431 | Ransyrt | ca 3600 BP | A | +/+ | + | - | - | +/- | R4 |
| R599 | Ransyrt | ca 3600 BP | A | +/+ | + | - | - | +/- | R4 |
| R576 | Ransyrt | ca 3600 BP | A | +/+ | - | - | - | +/- | R4 |
| R411A | Ransyrt | ca 3600 BP | B | +/+ | - | - | - | - | R0 |
| R411B | Ransyrt | ca 3600 BP | B | +/+ | - | - | - | +/- | R2 |
| R276 | Ransyrt | ca 3600 BP | C | +/+ | - | - | +/- | +/- | R6 |
| TB10-709 | Tilla Bulak | ca 3900 BP | A | +/+ | - | - | +/- | +/- | R6 |
| TB10-683 | Tilla Bulak | ca 3900 BP | A | +/+ | - | - | - | - | R0 |
| TB08-310 | Tilla Bulak | ca 3900 BP | A | +/+ | - | - | - | - | R0 |
| TB10-689 | Tilla Bulak | ca 3900 BP | A | +/+ | + | - | - | - | R1 |
| TB10-831 | Tilla Bulak | ca 3900 BP | A | +/+ | + | - | - | +/+ | R4 |
| TB08-266 | Tilla Bulak | ca 3900 BP | A | +/+ | + | - | - | +/- | R4 |
| TB10-897 | Tilla Bulak | ca 3900 BP | B | +/+ | + | - | - | +/- | R4 |
| TB10-792 | Tilla Bulak | ca 3900 BP | B | +/+ | - | - | - | +/+ | R2 |
| TB09-714 | Tilla Bulak | ca 3900 BP | C | +/+ | - | - | - | - | R0 |
| TB10-657 | Tilla Bulak | ca 3900 BP | D | +/+ | + | - | +/- | +/- | R7 |
| TB09-716 | Tilla Bulak | ca 3900 BP | D | +/+ | - | - | - | - | R0 |

1. Cai, D.-W.; Han, L.; Zhang, X.-L.; Zhou, H.; Zhu, H. DNA analysis of archaeological sheep remains from China. *J. Archaeol. Sci.* **2007**, *34*, 1347–1355, doi:10.1016/j.jas.2006.10.020.
2. Geörg, C. *Paläopopulationsgenetik von Schwein und Schaf in Südosteuropa und Transkaukasien*; VML: Stellerloh, Germany, 2013.
3. Peng, M.-S.; Fan, L.; Shi, N.-N.; Ning, T.; Yao, Y.-G.; Murphy, R. W.; Wang, W.-Z.; Zhang, Y.-P. DomeTree: a canonical toolkit for mitochondrial DNA analyses in domesticated animals. *Mol. Ecol. Resour.* **2015**, *15*, 1238–1242, doi:10.1111/1755-0998.12386.
4. Arnaud, F.; Caporale, M.; Varela, M.; Biek, R.; Chessa, B.; Alberti, A.; Golder, M.; Mura, M.; Zhang, Y.; Yu, L.; Pereira, F.; DeMartini, J.C.; Leymaster, K.; Spencer, T.E.; Palmarini, M. A Paradigm for Virus–Host Coevolution: Sequential Counter-Adaptations between Endogenous and Exogenous Retroviruses. *PLoS Pathog.* **2007**, *3*, e170, doi:10.1371/journal.ppat.0030170.

Chessa, B.; Pereira, F.; Arnaud, F.; Amorim, A.; Goyache, F.; Mainland, I.; Kao, R.R.; Pemberton, J.M.; Beraldi, D.; Stear, M.J.; et al. Revealing the History of Sheep Domestication Using Retrovirus Integrations. *Science* **2009**, *324*, 532–536, doi:10.1126/science.1170587.
